# Alzheimer Disease Classification through Transfer Learning Approach

**DOI:** 10.3390/diagnostics13040801

**Published:** 2023-02-20

**Authors:** Noman Raza, Asma Naseer, Maria Tamoor, Kashif Zafar

**Affiliations:** 1 Department of Computer Science, National University of Computer and Emerging Sciences, Lahore 54770, Pakistan; 2 Department of Computer Science, Forman Christian College, Lahore 54600, Pakistan

**Keywords:** gray matter, convolutional neural network, Alzheimer’s disease classification, dense-net

## Abstract

Alzheimer’s disease (AD) is a slow neurological disorder that destroys the thought process, and consciousness, of a human. It directly affects the development of mental ability and neurocognitive functionality. The number of patients with Alzheimer’s disease is increasing day by day, especially in old aged people, who are above 60 years of age, and, gradually, it becomes cause of their death. In this research, we discuss the segmentation and classification of the Magnetic resonance imaging (MRI) of Alzheimer’s disease, through the concept of transfer learning and customizing of the convolutional neural network (CNN) by specifically using images that are segmented by the Gray Matter (GM) of the brain. Instead of training and computing the proposed model accuracy from the start, we used a pre-trained deep learning model as our base model, and, after that, transfer learning was applied. The accuracy of the proposed model was tested over a different number of epochs, 10, 25, and 50. The overall accuracy of the proposed model was 97.84%.

## 1. Introduction

Alzheimer’s disease (AD) is a slow neurological disorder that destroys the thought process, and consciousness, of a human. It directly affects the development of mental ability and neurocognitive functionality [1]. AD is more common among older people, and sometimes it becomes the primary cause of their deaths [2,3]. People who have other medical problems at later age, such as diabetes, cardio-problems, and hypertension [1], are at higher risk of developing Alzheimer’s disease. There is no complete treatment for AD disease. However, early detection of this disease can help in taking preventative action at an early stage and can improve the symptoms of AD [4]. It is a primary reason for dementia in the elderly, due to the destruction of neurons related to the human memory [4], and to questioning, and learning functions [5]. This neurological disorder starts with slow deterioration and symptoms worsen with each passing day [6]. Memory issues are generally one of the first symptoms of AD [2], although preliminary symptoms may also vary from individual to individual [3]. Changes in different factors of thinking, inclusive of locating the proper words, spatial issues, and impaired judgment, might also become the early symptoms of AD [7]. The initial stage of mild cognitive impairment (MCI) is an early signal that a human may be an AD patient [8]. The number of AD patients is increasing day by day and it is expected that one out of eighty-five humans could be suffering from Alzheimer’s disease by 2050 [9,10].

The progression of Alzheimer’s disease can be diagnosed using clinical measures, but it is a very time-consuming process and expert persons are required to detect the symptoms [11,12]. Early diagnosis is very difficult to assess by an expert person, unless symptoms become very obvious. Early detection of AD can assist in lowering the risk of neuron disorder [13,14]. Early diagnosis can ensure that the patient is aware of the need to take precautionary measures to lower the risk of advancement of the disease from MCI to AD [1]. In recent times, different machine learning and deep learning methods have been proposed for the prediction of the stages of AD through self-regulating analysis of magnetic resonance imaging (MRI) images, which provide efficient and improved diagnosis results for AD [14,15,16]. The major factors, or parameters, that researchers use are the cortical thickness of the human brain, gray matter (GM) density in the brain, ventricle expansions, and brain shrivel. Many research studies claim that a correlation exists between grey matter reduction and certain brain diseases, like Alzheimer’s disease [13]. The hippocampus is the part of the brain that is affected at the initial stage of Alzheimer’s disease. White matter (WM), gray matter (GM), and cerebrospinal liquid are the major and most primitive tissues in human brain images. Out of these three fundamental tissues of the brain, researchers have discovered that GM shrivel corresponds more with corporeal diminishment in mild cognitive impairment [17].

Different statistical and machine learning methods, such as Support vector machine (SVM) [18] are used for automated recognition of Alzheimer’s disease. Lately, deep learning techniques, such as CNN and sparse auto-encoders, have surpassed the SVM learning techniques [11]. There are some limitations to using deep learning methods. The training from scratch of deep learning models requires a lot of computation and a large dataset of annotated medical images [19,20]. The collection of such a massive amount of medical images is a bottleneck for researchers. To overcome issues of large dataset requirements and computational issues associated with complex methods, the transfer learning approach can be used for different medical imaging modalities. Transfer learning is very useful, even for cross-domain applications, such as the fact that a model trained on images of natural things could be used for medical images, like X-rays, CT scans, MRIs, and many others [21]. The idea behind transfer learning is to utilize a pre-trained model on a new, small image, dataset of a different nature [22,23]. Transfer learning is an emerging deep learning technique in which an architecture devised for one task is reused as the initial point for a second task. The important motive behind transfer learning is the saving of knowledge attained while resolving a specific dilemma, followed by applying that gained knowledge to solve different problems.

**Figure 1:**
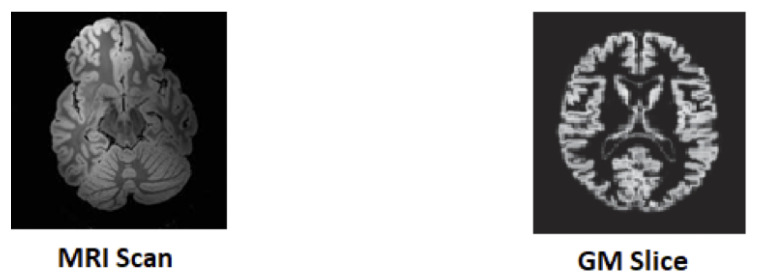
MRI to 2D GM slice.

In this research, a CNN-based transfer learning technique is used for the classification of neuro–medical AD scans into the following four categories: Alzheimer’s disease (AD), late mild cognitive impairment (LMCI), mild cognitive impairment (MCI), and normal cognition (NC). The elemental motivation for using transfer learning was to forward the features extracted from natural brain images to AD images, and to investigate a new approach for the categorization of AD that could be helpful for clinicians in proper diagnosis and decision-making. In this way, the patient can be guided in taking precautionary measures to lower the risk of MCI progressing to AD. The main objective is to produce improved results, even when using a small-sized dataset. Transfer learning gives us the flexibility to drastically reduce training time by reusing pre-trained models for new data and for obtaining better results without over-fitting. The proposed model uses a deep learning architecture as a base model where the last few convolutional layers are retained and then merged with fully connected layers. By applying the transfer learning approach with retraining of the last two layers, promising results are obtained on multi-class categorizations, such as AD, LMCI, EMCI, MCI, and NC. This research is primarily based on two-dimensional Gray matter (GM) images of the human brain, that are extracted from raw Magnetic resonance images (MRIs), these being more helpful in the detection of the early stage of AD. As illustrated in Figure 1, the MRI scans of the patients are converted to 2D slices in the pre-processing step, where the GM slices are focused for the extraction and training of the proposed model.

The major research contributions are mentioned below:

- A customized convolutional neural network with transfer learning is proposed for the classification of Alzheimer’s disease.
- A new corpus, consisting of four different types of AD, is developed. Each type consists of 1254 images.
- Extraction of 2D GM slices, using “SPM12”, which is very familiar in medical image pre-processing.
- Higher accuracy 97.84%, with a lower number of epochs, is achieved for the multi-class classification of AD.

The rest of the paper is organized in such a way that Section 2 describes the background of the techniques used in this research. Section 3 provides a detailed view of the related work, in which some related techniques of AD classifications are discussed. Section 4 describes the methodology and details of the experimental results. Section 5 elaborates the discussions, while Section 6 contains the conclusion of the research and future directions.

## 2. Background

Early diagnosis of Alzheimer’s disease can assist in lowering the peculiar disorder of the neurons. In this research, a model that takes a 2D GM slice as input to classify the stage of AD is discussed. A brief introduction for each of the basic concepts used in this research is provided below.

### 2.1. Convolutional Neural Network

Among many algorithms that enable a machine or a computer to train, perceive and learn for different medical classification problems, one is the convolutional neural network (CNN) [11,15,16]. CNN has revolutionized machine learning and artificial intelligence domains to make them akin to human brains. One of the important features of CNN is its characteristics of learning the features of images with training. In the human brain, the neurons are attached to each other in a specific pattern. CNN models are designed in the same manner. CNN models are multi-layered structures that perform in a group. The design of CNN comprises the following four kinds of layers [24]:

- Convolution
- Pooling
- Fully Connected
- Softmax

### 2.2. Transfer Learning

Transfer learning is an emerging deep learning technique in which an architecture trained for one task is reused as the initial point for a second task. The basic motivation behind this concept is to reduce training time, and to overcome issues associated with the need for large training sets. Pre-trained models are used for new problems without compromising the performance results.

### 2.3. Gray Metter

Gray matter and white matter are the two major tissues of which the central nervous system is composed [2,3]. Gray matter is the main part of the brain that is used to process information sent in the form of signals by the sensory organs of the human body.

### 2.4. DenseNet

**Figure 2:**
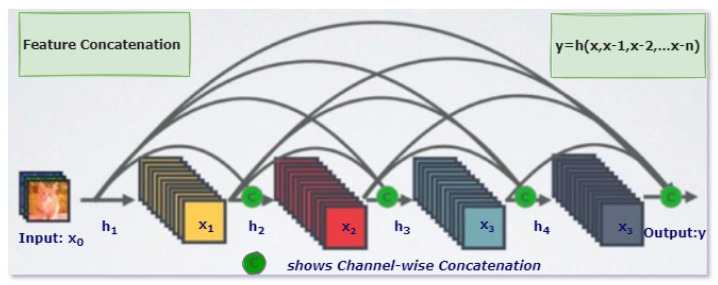
One Dense Block in DenseNet [26].

DenseNet is an efficient variant of a convolutional neural network, in which each layer has extra inputs from all previous layers and forwards its very own characteristic maps to all the next layers. Due to concatenation, each layer in the DenseNet obtains complete and comprehensive knowledge from all previous layers. In this architecture, each layer obtains extra inputs from all preceding layers and passes on its feature-maps to all subsequent layers, as illustrated in Figure 2 [25]. Instead of summation, as applies to ResNet, layers are combined using concatenation. Due to this layer-wise propagation of knowledge, DenseNet can be thinner and have better computational performance [26].

## 3. Related Work

In the paper, [27] a transfer learning approach was introduced by using the two most famous deep CNN architectures (Inception and VGG16) with the already trained and fine-tuned weights of ImageNet data. Using a pre-trained model on ImageNet, the researchers trained the last fully connected layer with a small number of training MRI scans. To overcome the over-fitting of the small training dataset, image entropy was applied to MRI images, to extract the most informative portions. An OASIS cross-sectional dataset with 416 subjects was used in an experiment aimed at the binary classification of AD. Five-fold cross-validation was applied with an 80 percent and 20 percent split between training and testing in the fully connected layer retraining. To compare the results VGG16 was also trained from scratch, as well as with transfer learning. Due to the small training set, the VGG16 trained from scratch performed less well in terms of accuracy, 74.12%, while the VGG16 with transfer learning provided 92.3% accuracy. Finally, Inception V4 was used with transfer learning that provided promising results with 96.25% accuracy.

In the paper [28] CNN with LeNet-5 was utilized for the classification of the brain with AD and the normal brain, by using functional MRI 4D data. In the first step, the 4D data was transformed into 2D by using the neuroimaging packages Nibabel and OpenCV. Then, 2D images were labeled as AD vs NC. The LeNet model, based on CNN, was then used for the binary classification of the images. The results were compared with the famous support vector machine model and, in contrast to it, the proposed model provided better results, with 96.86% accuracy.

In the paper [29] a framework with the combination of sparse auto-encoders (SAEs) and a softmax logistic regression was used, along with autoencoders, to use unlabeled data. Two data sets, MR and positron emission tomography (PET) from the ADNI database, were used. The main target of this research was to use SAE for high-level feature selection in the unsupervised pre-training stage. As a result of two different neuroimaging modalities, a zero-masking technique was used for the extraction of complimentary details from these different datasets. Features extracted from SAE, using unsupervised data, were then manipulated with a softmax regression. The performance of the model was tested on the classification of AD. In comparison with other advanced models, like SVM and other deep learning methods, the proposed model performed very well with 91.4 percent accuracy just because of its capability to extract features in one setting and its requiring of less labeled data.

In the paper, [30], a customized 2D-CNN model, with 9 depth-wise separable convolutional and normalization layers, was used, along with Inception V3 and Xception models for transfer learning. In this research, the classification of AD patients, class imbalance, and data leakage issues were discussed. The second fully connected layer used the sigmoid function as an activation function to categorize the data into two classes. An OASIS dataset, with T1-weighted structural MRI images, was used and the dataset was divided into 3 portions: set 1, set 2, and set 3. Cross-validations with 2-folds, 5-folds, and 9-folds were applied to the partitioned datasets, respectively. Dataset 1 was used for the prediction of AD, dataset 2 was used for class imbalance and dataset 3 was used for data leakage problems. For the AD classification on dataset 1, 45 subjects were used for training and validation purposes. Stochastic gradient descent (SGD) was used as an optimization algorithm. For loss function, binary cross entropy was used. In comparison with other deep learning models, the proposed model, which was based on transfer learning, provided promising results.

In another paper, [31], a deep learning framework with a softmax output layer and stacked autoencoders are used for the detection of Alzheimer’s disease and its initial stage MCI. MRI data of 311 patients available on the ADNI database was used. Gray matter (GM) was extracted from the MRI images, which made the baseline for the detection of MCI and the CMRGlc patterns using PET. Elastic Net is then used to extract the high-level features. In individual cross-fold, 90 percent of subjects are used for training and the rest of the 10 percent for testing. SK-SVM and MK-SVM are considered for comparison with the proposed model. The model gives 87.76 % accuracy in the binary classification of AD patients.

In another piece of research, [32] AlexNet, a fine-tuned pre-trained CNN, was used for the binary and multi-class classification of 3D MRI images. The proposed model was trained on the already pre-processed data in which WM, GM, and CSF were segmented and, then, the testing of the model was conducted on the unsegmented 3D MRI scans of the human brain. An OASIS dataset, consisting of 382 subjects, was used for training and testing. After the training of the proposed model on the segmented dataset, the retrained convolutional neural network was then used for the validation over the unsegmented 3D MRI images. For multi-class classification, the proposed model outperformed the binary classification, with 92.8% accuracy versus 89.6%.

In another paper [33], a modified Siamese CNN model, inspired by Oxford Net (VGG16), was used for the classification of AD stages. The basic idea behind the proposed model was to use the augmentation technique, with an extra convolutional layer in VGG16. Augmentation was applied to an OASIS dataset after the pre-processing phase. Two parallel layers of modified VGG16 worked for the extraction of the most important features. Batch normalization was applied to increase the learning rate, which gradually decreased, due to changing the parameter in individual layers of the CNN model. In comparison with the other state-of-the-art models, the proposed model provided 99.05 % accuracy, and it also reduced the problems of over-fitting and regularization. In [34], a layer-wise transfer learning approach and tissue segmentation were used for the classification of AD. The dataset used in this research was collected from the ADNI database. In the pre-processing step, the skull stripping, and extraction of GM, WM, and CSF were conducted using SPM12. The VGG-19 network was customized by modifying the last two fully connected and classification layers. Instead of freezing the trained fully connected layers, the researchers divided the model into two groups and then they gradually fixed CNN layers in different blocks. The training of the proposed model was done on both augmented and non-augmented datasets. In the first group, 8 CNN layers with 3 max-pooling layers were kept fixed, and in the second group 12 CNN layers along with 4 max-pooling layers were kept fixed. In experiments after the augmentation, the classification results of the proposed model were 98.73%, 83.72%, and 80% on AD vs NC, EMCI vs LMCI, and other classes, respectively.

In another paper [14], a cross-model technique, using the transfer learning technique, was used to reduce the over-fitting problem, while the training was done on a small set of MRI images. The proposed model was trained on the structural MRI data collected from the ADNI database and then tested on the DTI dataset. The outcome of the model on the two different cross-modalities was outstanding, with 92 percent accuracy on NC vs AD, 80 percent on NC vs MCI, and 85 percent on MCI vs AD.

In [16], the deep learning models, GoogLeNet and ResNet, were trained from scratch on structural MRI data sets available on the ADNI database. The main target of this research was to segment the gray matter (GM) and then train the CNN networks on these segmented GM images. The addition of the augmentation layer proved to be a useful step in the classification of the four stages of AD.

In another research [35], a convolutional network, with some extra parameters like gender, Mental-state exam score, and age, were trained on different datasets. The first data set was composed of clinically diagnosed Alzheimer’s patients and the second data set was extracted from the ADNI. The validation of the model was done on three different datasets, which included Australian Imaging, Biomarker and Lifestyle Flagship Study of Ageing, and the National Alzheimer’s Coordinating Center. The outcome of the model on multi-modal datasets was good in comparison to the other CNN models.

In a paper [36], ResNet18 is used for the classification of AD stages. The main purpose of this research was to utilize the Resting-state fMRI data, extracted from the ADNI database, which is a very useful neuroimaging technology used for the observation of neurodegenerative diseases. The concept of transfer learning was applied to the convolutional model, which was trained from scratch. The results of the proposed model were extracted with and without augmentations. They were also compared with other advanced CNN models. The outcomes of the proposed model showed promising results in the classification of AD stages.

## 4. Methodology

**Figure 3:**
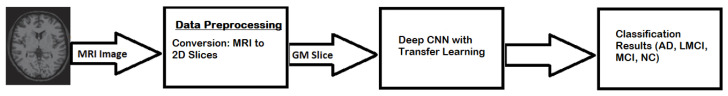
Proposed model workflow.

In this research, the segmentation of Alzheimer’s MRI images and their multi-class classification through transfer learning is proposed. For Alzheimer’s detection through MRI, Convolution Neural Network (CNN) was customized by using the specific GM-segmented images of the brain. Instead of training the model from scratch, a pre-trained deep learning model, Densenet-169, was used as a base model. Gradually, transfer learning was applied to this base model for Alzheimer’s detection. An overview of the proposed methodology is illustrated in Figure 3 which shows how MRI images were pre-processed and GM slices extracted. These GM slices were used for training the pre-trained deep learner and, finally, multi-class classification was performed.

### 4.1. DataSet

The dataset [34], used in this research was acquired from the publicly available Alzheimer’s Disease Neuroimaging Initiative (ADNI) database [37]. In 2004, this dataset was collected to analyze multiple sensitive techniques on various scanned images, like PET scan, sMRI, MRI and fMRI, etc. To evaluate the initial stages of Alzheimer’s disease [5] this dataset is considered a benchmark dataset. It contains images of four categories i.e., Alzheimer’s Disease (AD), Non-Cognitive (NC), Late Mild Cognitive Impairment (LMCI), and Mild Cognitive Impairment (MCI). Each category has 1254 images in total.

### 4.2. Data Preprocessing

**Figure 4:**
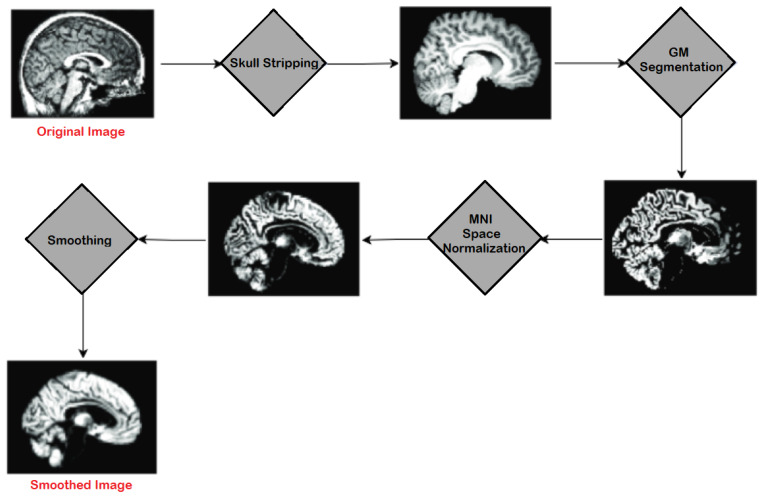
Major Steps of Pre-processing on MRIs.

In preprocessing, a series of steps were applied to the T1 weighted MRI images extracted from the publicly available database ADNI. For pre-processing, Statistical Parametric Mapping 12 (SPM12) was used which is quite often used in practice for medical and natural images. All the MRI images which were in Neuroimaging Informatics Technology Initiative (NIfTI) format were pre-processed to identify and extract the Gray Matter (GM) slices. The proposed model was trained on GM to detect and examine the initial changes in AD patients. The major steps of pre-processing encompassed segmentation, skull stripping, spatial normalization, and re-scaling. All the major steps, performed in the pre-processing of MRI images, are illustrated in Figure 4.

#### 4.2.1. Skull Stripping

For the morphometric analysis and study of brain MRIs, a preliminary process of skull stripping was performed, in which the tissues of the brain, i.e., cerebellum and cortex, were segmented from the surrounding zone consisting of non-brain and skull area. Among the multiple automated skull stripping approaches [38], the most commonly used technique, histogram thresholding, was followed by the employment of certain morphological processes [39]. The morphological procedures included erosion, and were based on anisotropic filters. The Snake method of contouring was also applied, to remove the eyes. The gradual process of skull stripping involved background and noise removal, followed by contour identification of the brain and, finally, refinement of the brain contour.

Head segmentation required noise removal, which was performed with the help of a threshold level, considering that the maximum noise was generated by Rayleigh probability distribution. The noise was removed by applying Equation (1):

(1) $$Rnoise\left(f\right)=\frac{f}{\sigma^{2}}exp\left(\frac{-f^{2}}{2\sigma^{2}}\right)$$

where *f* is the intensity of noise and σ is standard deviation of Rayleigh noise.

After that, the best-fit Rayleigh curve was subtracted from the histogram volume, as given in Equation (2):

(2) G(f)=h(f)−r(f)

where h(f) is histogram volume and r(f) is the best-fit curve. The value for r(f) was determined by minimizing error as given in Equation (3):

(3) $$\varepsilon_{\tau }=\sum_{f=0}^{\tau -1}g\left(f\right)+\sum_{f=\tau }^{\infty }r\left(f\right)$$

The said procedure produced speckle, which was outside the head region. This error was removed by using morphological operations in a 5×5 kernel.

The initial brain mask was generated in three steps. The first one was the smoothing of the brain and attenuation of non-brain regions for which anisotropic diffusion, given in Equation (4), was used in a nonlinear way:

(4) $$\frac{\partial }{\partial t}M\left(x,t\right)=▿.\left(C\left(x,t\right)▿M\left(x,t\right)\right)$$

where *M* is MRI, *x* represents its coordinates, *t* is the iteration step and C(x,t) is the function for diffusion, given in Equation (5).

Next, the MRI volume was defused with the help of automatic thresholding, and a diffusion function, given in Equation (5), was used to produce a binary mask:

(5) $$C\left(x,t\right)=exp(-{\left(\frac{\left|▿M(x,t)\right|}{\sqrt{2}f}\right)}^{2})$$

where *f* was constant for diffusion, which was set as 128.

Finally, non-brain regions were removed by using spatial and morphological information obtained from the head mask. All the holes within the mask region were filled by applying binary erosion with kernel size 10 × 10. As a result, the kernel separated the eyes from the brain. After erosion, all the regions where the centroid was outside the bounding box were eliminated, which was decided after applying heuristics. After erosion, binary dilation was applied with the same size as the kernel that was used for erosion. This helped in recovering the darkest pixels which were eliminated from the edge of the brain, due to threshold.

**Figure 5:**
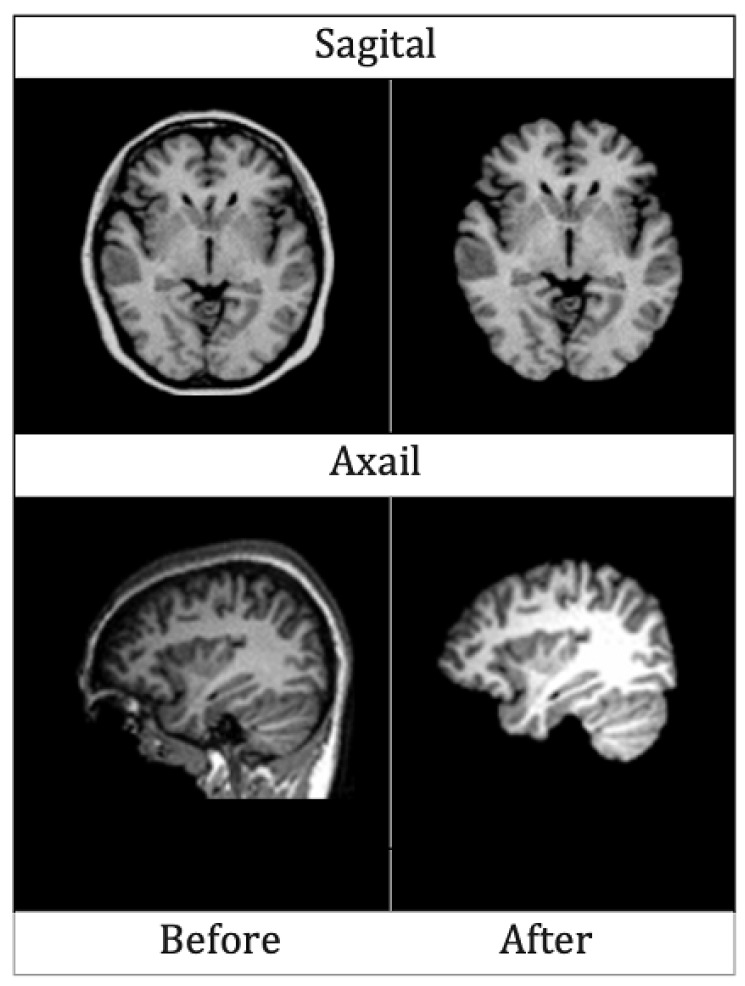
MRIs of Axial and Sagittal planes before and after skull striping.

Two sample images before and after skull stripping are given in Figure 5 for axial and sagittal planes.

#### 4.2.2. Segmentation

Segmentation is one of the major steps of pre-processing. For the diagnosis of certain brain disorders, segmenting brain tissues in Gray Matter (GM), White Matter (WM) and Cerebrospinal Fluid (CSF) makes the diagnosis process efficient.

**Figure 6:**
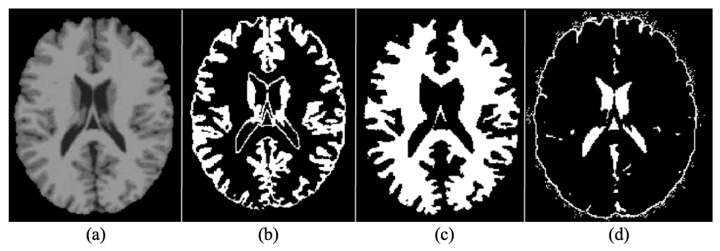
(**a**) Original (**b**) Grey Matter (GM (**c**) White Matter (WM) (**d**) Cerebrospinal Fluid.

The human brain MRI needs to be segmented into three main parts i.e., WM, GM, and CSF [40]. MRIs are mainly generated with the help of T1 and T2 weighted scan sequences. The main differences between the two are in Repetition Time (TR) and Time to Echo (TE), which is shorter for T1 and longer for T2. The contrast and brightness of MRIs are determined by T1 and T2, which affect the intensity levels of GM, WM, and SCF. For segmenting brain MRIs in GM, WM and SCF intensity probability distribution (IPD) was used, along with bias regularization, which was set to an extremely small regularization value i.e., 0.0001. Cutoff of 60 mm was used as Full Width at Half Maximum (FWHM), which was considered the width of a line structure at half of its highest amplitude. Figure 6 illustrates the results of segmenting an original brain MRI into GM, WM and SCF.

#### 4.2.3. Normalization

Spatial normalization is a process of mapping images containing gray matter to the same reference space. In this research, Voxel-based morphometry [41] was employed for spatial normalization. In Neuroanatomy, Voxel-based morphometry measures the dissimilarity between local tissue clusters of the brain and several other brain images. The differences are calculated via a voxel-wise comparison between the two brain images and the images are mapped to a reference brain space. For this purpose, the Montreal Neurological Institute (MNI) space was used to identify the boundaries around the brain.

#### 4.2.4. Rescaling

After the segmentation of raw MRI images, 256×240 shaped data samples were extracted. All these images were then re-scaled to 224×224, that were, finally,. used to train, test, and evaluate the proposed model. The re-scaling adjusted numbers for comparing values even if they were out of scope. Furthermore, it reduced the number of trainable parameters of deep learners and they trained faster.

#### 4.2.5. Smoothing

**Figure 7:**
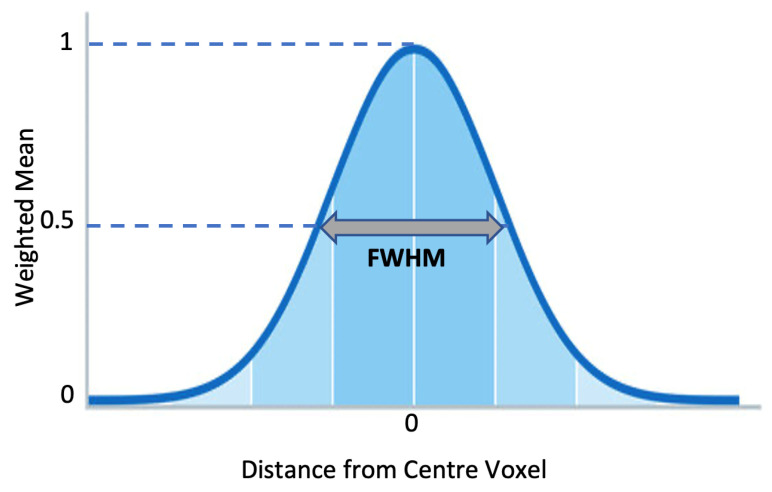
Gaussian Smoothing Kernel.

After rescaling, MRIs were smoothed by applying a 3D Gaussian kernel with voxel size (2 2 2). Each voxel was updated by the weighted mean value, attained from the adjacent voxels. The weight was decided based on the Gaussian shape that surrounded the voxel. The variance or width of the Gaussian curve determined how much smoothing was applied. With higher variance, more neighboring values were considered in calculating the mean value, which was expressed as FWHM. Figure 7 illustrates the Gaussian Kernel.

#### 4.2.6. Augmentation

**Figure 8:**
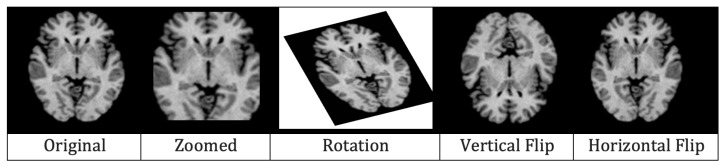
Effects of augmentation on MRI.

The last step of data pre-processing was augmentation which was adopted to avoid over-fitting. For generating augmented MRIs, five augmentation techniques, i.e., rescaling, rotation, zooming, horizontal flip, and vertical flip, were applied. In re-scaling, images were re-scaled to a range between 0 and 1 by multiplying each cell with $\frac{1}{255}$. The MRIs were rotated at 30${}^{\circ }$ in an anti-clockwise manner. All the MRIs were also zoomed in with a ratio of 0.2. Finally, vertical and horizontal flips were applied and, in this way, five images in total were generated from one pre-processed MRI. Figure 8 illustrates the results of augmentation.

### 4.3. Architecture

**Figure 9:**
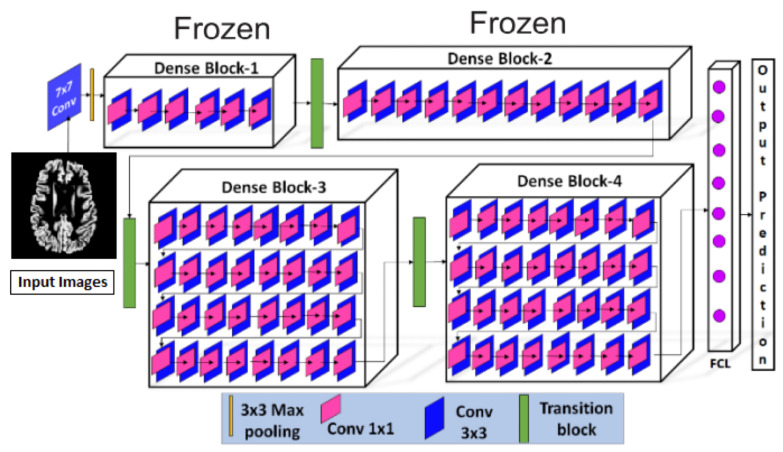
Proposed Model Architecture.

After pre-processing, the pre-possessed images were provided to the proposed Convolutional Neural Network, shown in Figure 9. The proposed CNN model consisted of a 7 × 7 convolution layers, 3 × 3 max-pooling layers, four dense blocks with a different number of convolution layers (6, 12, 32, 32) in respective blocks, three transitional layers, and a fully connected classification layer. Three major components, i.e., the convolution layer, dense blocks, and transition layers, were used to extract the features from the pre-processed MRIs. The convolution layer with a kernel matrix of size 7 × 7 and a stride value set as 2, was used for the extraction of macro-features [42]. These extracted macro-level features were then forwarded to dense blocks, where more features were extracted. Each dense block contained 1 × 1 convolutions, which were used to reduce the number of input feature maps, followed by 3 × 3 convolution layers. In each dense block, the extracted features were passed to all the next layers. Between the dense blocks, there were transition layers, along with the activation functions, like batch normalization and ReLu, etc. The transition layer contained 1 × 1 convolution and a 2 × 2 max pooling layer with a stride value set as 2. Once the transition layer reduced the dimensions of the features, they were forwarded to the fully connected classification layer where Softmax was used for the classification. Initially, all the initial blocks of layers were kept frozen and the last two blocks were retrained.

The details of all hyperparameters are listed in Table 1.

The concept of dense connectivity used in this research is represented in Equation (6):

(6) $$\begin{array}{c} c_{l}=H_{l}\left[\left(c_{0},c_{1},c_{2},\ldots \ldots c_{l-1}\right)\right] \end{array}$$

where $H_{l}$ is the non-linear transformation of $L^{th}$ layer and $\left[c_{0},c_{1},c_{2},c_{3}....\right]$ are the feature maps forwarded by all the previous layers to the $L^{th}$ layer.

The growth rate at the $L^{th}$ layer, which revealed how much information was added by the previous layers, was calculated via Equation (7):

(7) $$\begin{array}{c} g_{l}=\left[g_{0}+g\times \left(l-1\right)\right] \end{array}$$

where the hyper parameter *g* is the growth rate.

Convolutional layers were used to apply the filter on the input images to extract the feature maps. Once the feature maps were extracted by applying different filters, they were passed through the ReLU activation function. To obtain the non-linear input to the unit $M_{ij}^{l}$, all the previous layers’ benefaction were summed up, as described in Equation (8):

(8) $$\begin{array}{c} M_{ij}^{l}=\sum_{a=0}^{m-1}\sum_{b=0}^{m-1}\mu abY_{\left(i+a)(j+b\right)}^{l-1} \end{array}$$

To reduce the dimensionality of the extracted feature maps, the max pooling layer was used. Considering the dimensions of feature maps as $\left[m_{h},m_{w},m_{c}\right]$, representing height, width, and channels of the feature maps, the dimensions were reduced by applying Equation (9) and resultant feature maps were achieved.

(9) $$\begin{array}{c} max_{p}=\frac{\left(m_{h}-f+1\right)}{s}\times \frac{\left(m_{w}-f+1\right)}{s}\times m_{c} \end{array}$$

where *f* represents the size of the filter and *s* represents the strides.

In the dense layers, a neuron received the input from all the neurons of its preceding layer to perform the matrix multiplication. The standard equation for the matrix multiplication in the dense layer is shown in Equation (10):

(10) $$\begin{array}{c} N.\lambda =\left(\begin{array}{ccccc} n_{11} & n_{12} & ..... & n_{1y} & p_{1}\\ n_{21} & n_{22} & ..... & n_{2n} & p_{2}\\ \vdots & \vdots & & \vdots & \vdots \\ \vdots & \vdots & & \vdots & \vdots \\ \vdots & \vdots & & \vdots & \vdots \\ n_{x1} & n_{x2} & ..... & n_{xy} & p_{y} \end{array}\right) \end{array}$$

where the N represents matrix dimensions of x×y and another matrix P with dimensions 1×y and λ is the matrix of the trained parameters of the previous layer.

The λ, representing the matrix of the trained parameters of the previous layer, was updated by using the back-propagation in the training phase. The back-propagation was used to adjust the weights $w_{k}$ associated with a layer *k* over the learning rate η, as shown in Equation (11):

(11) $$w^{k}=w^{k}-\eta \times dw^{k}$$

where *dw* is the partial derivatives of the loss function.

The partial derivative *dw* of the loss function of *w* was acquired by employing Equation (12):

(12) $$dw^{k}=\frac{\partial L}{\partial w^{k}}=\frac{1}{n}dZ^{k}A^{[k-1]T}$$

where $Z^{k}$ is the activation function and $A^{k}$ is the non-linear activation function at layer *k* [43].

**Figure 10:**
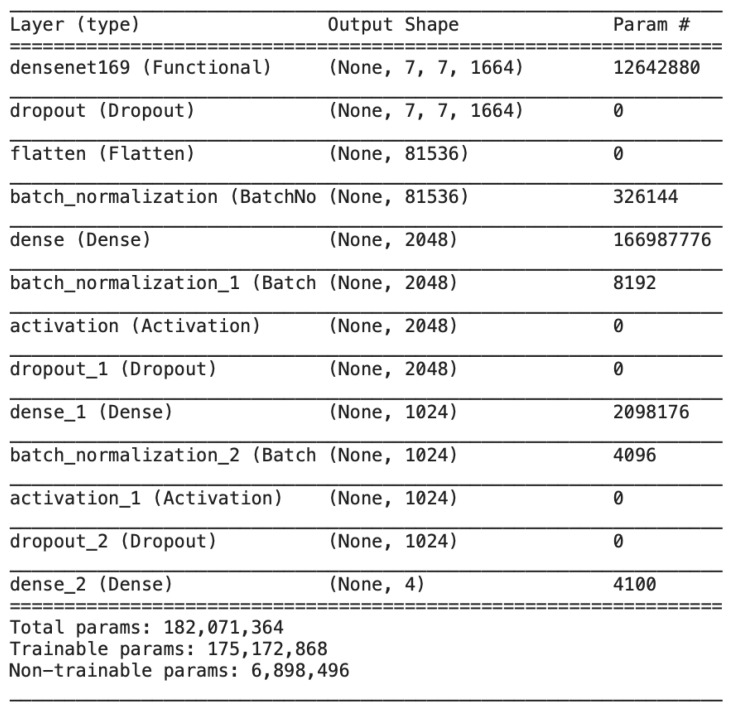
Output size and model parameters at each layer of the proposed DenseNet.

Figure 10 illustrates all the trainable and untrainable parameters of the proposed model, DenseNet, along with the output shape at each layer.

### 4.4. Experimental Setup and Training

For the performance evaluation for Alzheimer’s classification, four transfer learning-based models were trained with the same architecture, as illustrated in Figure 9. The first model was trained without using the feature of transfer learning and was provided with the images without augmentation. The second one was again trained without the feature of transfer learning but was provided with augmented MRIs. The next two models were used with the feature of transfer learning and were trained on only original MRIs and augmented MRIs, respectively.

**Figure 11:**
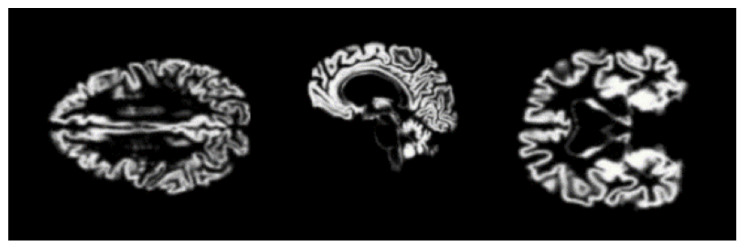
Gray matter images for proposed model.

**Figure 12:**
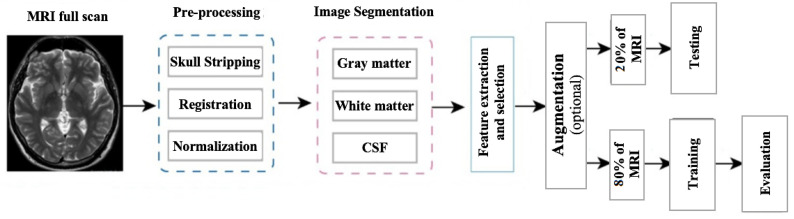
Framework of the proposed methodology.

Gray matter (GM) images, as shown in Figure 11, were provided to the models for training. Three views of these MRIs, i.e., axial, coronal, and sagittal, were considered for training the models and, in total, 1254 images from each category, i.e., AD, NC, MCI, and LMCI, were used. In two steps, the data were divided into training, validation, and testing sets. In the first step, 20% of the data were split and separated for testing. The remaining 80% of the data were further divided into training and validation sets, as explained in Figure 12.

During the training of the model, the number of epochs was gradually increased to verify the training accuracy and validation loss. The gradual increase in training accuracy and reduction in training loss with a different number of epochs is illustrated in Table 2.

## 5. Results

The four deep learning-based models were tested over the 20% data from the ADNI dataset. To check the performance of the proposed models, multiple measures, i.e., sensitivity, Equation (13), specificity, Equation (14), and accuracy, Equation (15), were used. All of these measures were elaborated in terms of True Positive (TP), False Positive (FP), False Negative (FN), and False Positive (FP) in their respective equations.

(13) $$\mathrm{Sensitivity}=\frac{\mathrm{TP}}{\mathrm{TP}+\mathrm{FN}}$$

(14) $$\mathrm{Specificity}=\frac{\mathrm{TN}}{\mathrm{TN}+\mathrm{FP}}$$

(15) $$\mathrm{Accuracy}=\frac{\mathrm{TN}+\mathrm{TP}}{\mathrm{TP}+\mathrm{TN}+\mathrm{FP}+\mathrm{FN}}$$

The class-wise outcomes of these measures are depicted in Table 3, while the confusion matrix of the four classes for the best achieved accuracy is given in Table 4 and Table 5. We can see that the proposed model performed well in the classification of all categories, especially in the case of AD vs NC, MCI, and LMCI, with higher accuracy of 99.68%, sensitivity of 99.65%, and specificity of 99.69%.

## 6. Discussions

**Figure 13:**
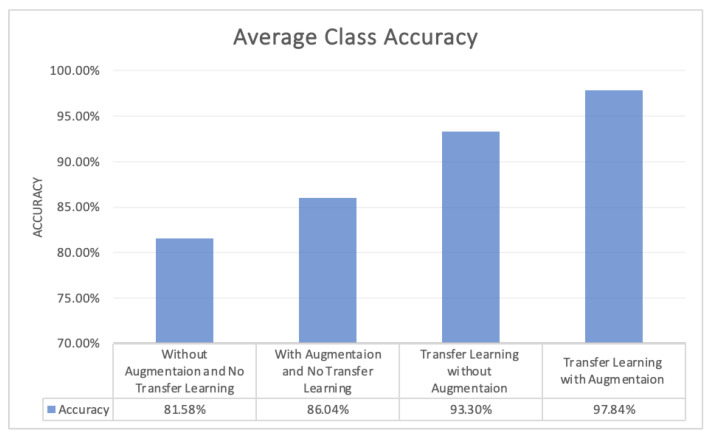
Average Class Accuracy of the four Models.

Numerous studies have been conducted recently on early diagnosis of Alzheimer’s disease by utilizing deep learning techniques, especially CNN. Most of the diagnosing systems are developed by training deep learning models from scratch and with a huge dataset that is trained over a large number of epochs. This study proposed a transfer learning assisted fine-tuning approach for the detection of four stages of Alzheimer’s disease. The designed model was evaluated by using prepossessed GM sliced images with a different number of epochs. The performance of the proposed was evaluated based on three measures, which were sensitivity, accuracy, and specificity. All of these three measures play a huge role in the correct classification of healthy versus ill persons. Gray matter (GM) is the main fundamental part of the human brain which processes information sent in the form of signals by different sensory organs of the body. GM is more helpful in the early diagnosis of AD. This research focused on the segregation of AD patients from healthy people. The proposed model was gradually trained and tested with a different number of epochs. Initially, the model was trained and tested over 10 epochs, with 93.11% accuracy. In the next phase, the number of epochs increased from 10 to 25, and then in the next phase the epochs increased from 25 to 50, with an overall accuracy of 97.84%, which elaborated the effectiveness of the proposed study (Figure 13).

Table 6 depicts the comparison with the base article [32] and the other studies that have contributed to the early diagnosis of Alzheimer’s disease. However, the proposed model performs outstandingly with 97.84% accuracy in the multi-class classification of AD with a very less number of epochs.

Early detection of Alzheimer’s disease, using MRIs, can be challenging, due to the presence of different artifacts. like noisy background, low contrast and partial volume [45]. To overcome these issues, more recent technologies, like Functional magnetic resonance imaging (fMRI), can be used in the future for the early detection of AD.

## 7. Conclusions

Alzheimer’s disease is a slow neurological disorder that destroys the thought processes, and consciousness, of a human and, mostly, the aptness to perform straightforward tasks. Many deep learning models have been proposed to detect and classify the stages of AD. Gary matter is the main fundamental part of the human brain that is mainly affected by Alzheimer’s disease and it plays a major role in the gradual destruction of neurons. In this research, a deep learning model, Dense-Net, with transfer learning, was applied to the MRI dataset. The main objective of this research was to classify the stages of Alzheimer’s disease, based on the extracted gray matter (GM), the main fundamental part of the human brain. MRI scans were segmented into 3 parts, GM, WM, and CSF, using the SPM12 for pre-processing. The 2D GM slices were used as input for the training and testing of the model. A pre-trained DenseNet model, with retraining of the last two blocks, was applied over the segmented GM slices. The proposed model provided promising results, with 97.84% accuracy in the multi-class classification of AD.

There were certain pros and cons of the proposed technique. DenseNet is well-reputed, due to its applications in medical imaging [46]. The analysis of medical images by DenseNet is remarkable, as it acquires a comprehensive and complete knowledge of image details from all previous layers. In addition to that, the propagation which takes place layer-wise can be made thinner to make it computationally economical without compromising accuracy, due to the fact that the final prediction depends on features obtained from all layers. As the proposed methodology was based on transfer learning, so the significant limitation of this approach was negative transfer, due to which, for initial training, the target and initial problems were supposed to be adequately homogeneous.

Still, considering the outstanding performance of the proposed model with respect to other CNN models, in the future, this model could be applied for the detection and classification of lungs, and other diseases.

## Figures and Tables

**Table 1:** Hyperparameters of the proposed model.

HYPERPARAMETERS	
Activation Function	ReLU
Epochs	50
Batch Size	128
Optimizer	Adam
Loss Function	Categorical Cross Entropy
Drop out	0.4

**Table 2:** Training accuracy and validation loss for transfer learning-based model, trained on augmented MRIs.

	Epochs 10	Epochs 25	Epochs 50
Loss	6.89%	3.18%	2.16%
Accuracy	93.11%	96.82%	97.84%

**Table 3:** Confusion Matrix.

	Predicted				
		**MCI**	**AD**	**NCI**	**LMCI**
**Actual**	**MCI**	248	1	1	0
	**AD**	1	247	1	1
	**NCI**	2	1	247	0
	**LMCI**	0	1	0	249

**Table 4:** Class-wise performance comparison of proposed model.

Image Classes	Specificity	Sensitivity	Accuracy
MCI vs. AD, NC and LMCI	99.89	98.42	99.52
LMCI vs. AD, NC and MCI	97.76	99.36	98.16
NC vs. AD, MCI and LMCI	99.78	94.32	98.33
AD vs. NC, MCI and LMCI	99.69	99.65	99.68

**Table 5:** Accuracy achieved by the four models with and without Augmentation and Transfer Learning.

Transfer Learning	Augmented Data	Accuracy
No	No	81.58%
No	Yes	86.04%
Yes	No	93.30%
Yes	Yes	97.84%

**Table 6:** Comparison of the proposed model with existing models.

Model	Year	Accuracy
Inception V4 [27]	2017	96.25%
AlexNet [32]	2019	92.80%
GoogLeNet [44]	2022	96.39%
ResNet-18 [15]	2021	96.88%
**Proposed Model**		**97.84%**

## Data Availability

The dataset [34], used in this research, is acquired from the publicly available Alzheimer’s Disease Neuroimaging Initiative (ADNI) database [37].
